# Examining Test-Retest Reliability and Reliable Change for Cognition Endpoints for the CENTER-TBI Neuropsychological Test Battery

**DOI:** 10.3389/fneur.2020.541533

**Published:** 2020-10-20

**Authors:** Jonas Stenberg, Justin E. Karr, Rune H. Karlsen, Toril Skandsen, Noah D. Silverberg, Grant L. Iverson

**Affiliations:** ^1^Department of Neuromedicine and Movement Science, Norwegian University of Science and Technology (NTNU), Trondheim, Norway; ^2^Department of Neurosurgery, St. Olavs Hospital, Trondheim University Hospital, Trondheim, Norway; ^3^Department of Physical Medicine and Rehabilitation, Harvard Medical School, Boston, MA, United States; ^4^Department of Psychiatry, Harvard Medical School, Boston, MA, United States; ^5^Spaulding Rehabilitation Hospital, Charlestown, MA, United States; ^6^Home Base, A Red Sox Foundation and Massachusetts General Hospital Program, Charlestown, MA, United States; ^7^Spaulding Research Institute, Charlestown, MA, United States; ^8^Department of Physical Medicine and Rehabilitation, St. Olavs Hospital, Trondheim University Hospital, Trondheim, Norway; ^9^Department of Psychology, University of British Columbia, Vancouver, BC, Canada; ^10^Division of Physical Medicine and Rehabilitation, University of British Columbia, Vancouver, BC, Canada; ^11^Rehabilitation Research Program, GF Strong Rehabilitation Centre, Vancouver, BC, Canada

**Keywords:** brain concussion, brain injury, cognition, neuropsychology, psychometrics

## Abstract

**Objective:** Seven candidate cognition composite scores have been developed and evaluated as part of a research program designed to validate a cognition endpoint for traumatic brain injury (TBI) research and clinical trials, but these composites have yet to be examined longitudinally. This study examined test-retest reliability and methods for determining reliable change for these seven candidate composite scores, using the neuropsychological test battery from the Collaborative European NeuroTrauma Effectiveness Research in Traumatic Brain Injury (CENTER-TBI).

**Methods:** Participants (18–59 years-old) with mild TBI (*n* = 124), orthopedic trauma without head injury (*n* = 67), and healthy community controls (*n* = 63) from the Trondheim MTBI follow-up study completed the CENTER-TBI neuropsychological test battery at 2 weeks and 3 months after injury. The battery included both traditional paper-and-pencil tests and computerized tests from the Cambridge Neuropsychological Test Automated Battery (CANTAB). Seven composite scores were calculated for the paper-and-pencil tests, the CANTAB tests, and all tests combined (i.e., 21 composites in total on each assessment): the overall test battery mean (OTBM); global deficit score (GDS); neuropsychological deficit score-weighted (NDS-W); low score composite (LSC); and the number of scores ≤5th percentile, ≤16th percentile, or <50th percentile. The OTBM was calculated by averaging *T* scores for all tests. The other composite scores were deficit-based scores, assigning different weights to low scores.

**Results:** All composites revealed better cognitive performance at the 3-month assessment compared to the 2-week assessment and the magnitude of improvement was similar across groups. Differences, in terms of effect sizes, were largest on the OTBMs. In the combined composites, the test-retest correlation was highest for the OTBM (Spearman's *rho* = 0.87, in the community control group) and lowest for the number of scores ≤5th percentile (*rho* = 0.41).

**Conclusion:** The high test-retest reliability of the OTBM appears to favor its use in TBI research; however, future studies are needed to examine these candidate composite scores in participants with more severe TBIs and cognitive deficits and the association of the composites with functional outcomes.

## Introduction

Cognitive impairment is a core clinical feature of traumatic brain injury (TBI) (1). In the mildest of TBIs, it might resolve within hours or days (2); and in severe TBI, it can be permanent and disabling (3, 4). Cognitive functioning is of considerable interest as an outcome measure in TBI research and clinical trials (5). Given that cognition is multifaceted and it is commonly measured with a variety of tests that index different cognitive domains, it would be useful to create a single composite score, or cognition endpoint, for TBI research and clinical trials (6). Our research team has recently examined seven candidate composite scores, derived from prior studies (7, 8), using the neuropsychological test battery from the Collaborative European NeuroTrauma Effectiveness Research in Traumatic Brain Injury (CENTER-TBI) (9). CENTER-TBI is a large-scale, multi-national, observational study that aspires to identify best practices, develop precision medicine, and improve outcomes for people with TBIs via comparative-effectiveness studies (10–12). In our prior study on the CENTER-TBI neuropsychological battery, data from the Trondheim MTBI follow-up study, in which the CENTER-TBI neuropsychological battery was administered, was used to calculate the composite scores separately for four traditional paper-and-pencil tests, five computerized neuropsychological tests, and a combined battery of all nine tests (9). Before determining which candidate composite score(s) might be most useful for clinical research in TBI, and with the CENTER-TBI battery in particular, it is important to examine these scores longitudinally for stability (in people without TBI) and sensitivity to change (in people with TBI). Test-retest reliability, in the present study, represents an estimate of the stability and consistency of neuropsychological test scores across two testing sessions. Test-retest reliability is influenced by the internal consistency of the test, measurement error related to time and situational variables (13), and normal variability in human cognition. Changes in cognitive scores from test to retest are related to several factors such as susceptibility to practice effects, measurement error, the test-retest interval between administrations, and regression to the mean. Moreover, person-specific factors can influence test to retest difference scores, such as initial level of performance, motivation, and effort. Some cognitive abilities can be measured precisely and reliably, such as a person's ability to read single words in his or her native and dominant language, whereas tests of other cognitive abilities, such as memory and executive functioning, usually have lower reliabilities (14) because people's test scores are more likely to be influenced by a variety of factors (e.g., practice effects, situational distractions, measurement imprecision, regression, and effort). A composite score with high test-retest reliability that is sensitive to changes in cognitive functioning coinciding with the natural history of TBI recovery is desirable in TBI research and clinical trials that use rate of change as the primary endpoint. The purpose of this descriptive study is to compare and contrast the test-retest reliabilities, and estimates of reliable change, for the seven candidate composite scores that have been recently applied to the CENTER-TBI battery (9) in patients with mild TBI, in trauma controls without head injury, and in healthy community controls.

## Methods

### Participants

The participants in the present study were part of the Trondheim Mild Traumatic Brain Injury (MTBI) Study (15). Patients with MTBI were recruited from April 2014 to December 2015. In the present study, patients were included if they were between ages 18 and 59 years and sustained a MTBI per the criteria described by the WHO Collaborating Center Task Force on Mild Traumatic Brain Injury: (a) mechanical energy to the head from external physical forces; (b) Glasgow Coma Scale (GCS) score of 13–15 at presentation to the emergency department; and (c) either witnessed loss of consciousness (LOC) <30 min, confusion, or post-traumatic amnesia (PTA) <24 h, or intracranial traumatic lesion not requiring surgery (16). Exclusion criteria were non-fluency in the Norwegian language; pre-existing severe neurological (e.g., stroke, multiple sclerosis), psychiatric, somatic, or substance use disorders, determined to be severe enough to likely interfere with follow-up; a prior history of a complicated mild, moderate, or severe TBI; or other concurrent major orthopedic trauma; or moderate/severe TBI.

Recruitment took place at a level 1 trauma center in Trondheim, Norway, and at the municipal emergency clinic, an outpatient clinic run by general practitioners. LOC was categorized as present if witnessed. Duration of PTA, defined as the time after injury for which the patient had no continuous memory, was dichotomized to either <1 h or 1–24 h. Intracranial traumatic findings were obtained from magnetic resonance imaging (MRI), performed within 72 h, previously described in detail (17). Two control groups were recruited. One group consisted of patients with orthopedic injuries, free from trauma affecting the head, neck, or the dominant upper extremity (i.e., trauma controls). The trauma controls were recruited from the same emergency departments as the patients with MTBI. Fractures to the upper extremities (35.8%), lower extremities (25.4%), and soft tissue injuries to the lower extremities (26.9%) were the most common injuries among the trauma controls. Injuries commonly occurred during sports or recreational activities (38.8%) and 25.4% had an injury requiring surgery. The other group consisted of healthy community controls, not receiving treatment for severe psychiatric disorder (e.g., bipolar or psychotic disorder). The community controls were recruited among hospital and university staff, students, and acquaintances of staff, students and patients. The study was approved by the regional committee for research ethics (REK 2013/754) and was conducted in accordance with the Helsinki declaration. All participants gave informed consent.

### Neuropsychological Assessment

Participants with MTBI underwent neuropsychological testing ~2 weeks (*M* = 16.6 days, SD = 3.2 days) and 3 months (*M* = 95.0 days, SD = 6.6 days) after the injury. The trauma controls were also evaluated 2 weeks (*M* = 17.1 days, SD = 3.5 days) and 3 months (*M* = 95.3 days, SD = 10.5 days) after injury. The community controls were tested ~3 months apart (*M* = 95.1 days, SD = 11.6 days). The tests were administrated by research staff with at least a Bachelor's degree in clinical psychology or neuroscience who were supervised by a licensed clinical psychologist. The testing involved a larger battery, with only the tests included in the CENTER-TBI neuropsychological battery analyzed in the current study. To calculate the composite scores (described below), normative data was required for each included outcome to convert raw scores into age-referenced *T* scores.

The traditional paper-and-pencil tests included in the CENTER-TBI battery are the Trail Making Test (TMT) Parts A and B and the Rey Auditory Verbal Test (RAVLT). In TMT Part A (18), participants connect numbered circles in order as fast as possible, and in TMT Part B, participant alternate between numbered and lettered circles, switching between connecting them in numerical and alphabetical order as fast as possible. For both TMT Parts A and B, the outcome measure was time-to-completion, with normative data from Mitrushina et al. (19) used to calculate age-referenced T scores. The RAVLT (18) involves participants listening to and recalling a list of 15 words over five trials, and then recalling these words again following the introduction of a distractor list and after a 20-min delay. The RAVLT outcome measures included in composite calculation were the total number of words recalled across the five learning trials and the total number of words recalled following the 20-min delay. The 2-week and the 3-month assessments involved different word lists for the RAVLT. Age-referenced T scores were calculated based on normative data from Schmidt (20) published in Strauss et al. (18).

The CENTER-TBI battery includes six tablet administered tasks from the Cambridge Neuropsychological Test Automated Battery (CANTAB): Attention Switching Task (AST), Paired Associates Learning (PAL), Rapid Visual Processing (RVP), Spatial Working Memory (SWM), Reaction Time Index (RTI), and Stockings of Cambridge (SOC). The CANTAB software generates age-referenced *T* scores for all of these tasks except for the AST (21), which was therefore not included in the present study (i.e., in total five CANTAB tests were included). Each CANTAB task generates multiple outcome measures. We selected one outcome measure for each task to be included in the composite score calculation based on the CANTAB's “Recommended Measures Report” (21). On the PAL visual memory task, participants briefly observe a series of boxes that contain different patterns. The patterns are then hidden, and the participant must match a target pattern to the box that contains that pattern. “Total errors” (adjusted for the number of trials completed) was chosen as the outcome measure, with more errors indicative of worse performance. On the RVP processing speed task, individual numbers appear rapidly on the screen (i.e., 100 presentations per minute) and participants respond to target sequences of digits presented in a specific order (e.g., 2-4-6). The outcome measure chosen was “A prime,” which measures discriminability between target and non-target sequences. A higher score is indicative of better performance. On the Spatial Working Memory (SWM) task, participants search through a series of boxes for a token. Once the token is found, a new token is hidden in one of the remaining boxes. A token is never hidden in the same box twice, and participants must remember where tokens were previously presented in order to avoid errors. “Between errors” was chosen as the outcome, which is the number of times a participant revisits a box in which a token was previously found. More errors are indicative of worse performance. On the Reaction Time Index (RTI) task, the participant responds as quickly as possible when a yellow dot appears in one of five white circles, with response time in milliseconds chosen as the outcome measure (shorter response time equals better performance). On the SOC executive function task, two displays with three balls presented inside stockings are presented. Participants move the balls in one display to produce an identical arrangement to the other display. The outcome measure was the number of problems solved with the minimum possible moves, with a higher score indicative of better performance.

### Composite Scores

Seven different composite scores, previously described in detail (7, 8), were calculated for the present study. Each composite score was calculated for the traditional paper-and-pencil tests only, the CANTAB tests only, and all tests (i.e., a combined composite). All raw scores were converted to age-referenced *T* scores (*M* = 50, SD = 10, in the normative sample), with higher scores indicative of better performance, before the composites were calculated.

The Overall Test Battery Mean (OTBM) was calculated by averaging T scores for all tests (22, 23). Lower scores indicate worse performance.

The Global Deficit Score (GDS) has been used in previous research (24, 25) and was calculated by assigning the following weights to T scores from each test: ≥40 = 0, 39–35 = 1, 34–30 = 2, 29–25 = 3, 24–20 = 4, and ≤19 = 5. Each participant's mean weight was then calculated for the entire batteries. Higher scores indicate worse performance.

The Neuropsychological Deficit Score-Weighted (NDS-W) is a new composite calculated in previous cognition endpoint research only (7–9). It assigns the following weights to T scores: ≥50 = 0, 49–47 = 0.25, 46–44 = 0.5, 43–41 = 1, 40–37 = 1.5, 36–35 = 2, 34–31 = 3, 30–28 = 4, 27–24 = 5, 23–21 = 6, and ≤20 = 7. The mean weight was then calculated for the entire batteries. Higher scores indicate worse performance. This new deficit score is similar to the GDS, but provides an increase in gradations to lower the floor effect of the GDS.

The Low Score Composite (LSC) is a new composite calculated in previous cognition endpoint research only (7–9). T scores of 50 or higher are assigned a weight of 50, and *T* scores below 50 are assigned a weight that equals the *T* score (i.e., a *T* score of 40 would equal a weight of 40). The mean weight was then calculated for the entire batteries. Lower scores indicate worse performance. This new composite score provides an even greater increase in gradation than the NDS-W.

The number of scores at or below the 5th percentile (#≤5th %tile) is calculated by assigning the value 1 to scores at or below the 5th percentile (*T* = 34) and a zero to scores above the 5th percentile. These values are then summed for each participant. Higher scores indicate worse performance. This score has been used in research calculating multivariate base rates for a range of neuropsychological test batteries (26–33).

The number of scores at or below the 16th percentile (# ≤ 16th %tile) is calculated by assigning the value 1 to scores at or below the 16th percentile (*T* = 40) and a zero to scores above the 16th percentile. These values are then summed for each participant. Higher scores indicate worse performance. This score has also been calculated in previous multivariate base rate research (26–33).

The number of scores below the 50th percentile (#<50th %tile) is a new composite score, inspired by research on multivariate base rates, and previously calculated in cognition endpoint research only (7–9). It is calculated by assigning the value 1 to scores below the 50th percentile (*T* score 49) and a zero to scores at or above the 50th percentile. These values are then summed for each participant. Higher scores indicate worse performance.

### Statistical Analyses

Wilcoxon Signed-Rank Tests were used to evaluate differences in the composite scores between the assessments, with *r* reported as the effect size (i.e., the *z*-statistic associated with the Wilcoxon Signed-Rank Test divided by the square root of the sample size) (34, 35). This effect size can be interpreted as: 0.1 = small, 0.3 = medium, 0.5 = large (36). Cohen's *d*s [the mean difference between the assessments divided by the pooled standard deviation from the two assessments (37)] are also reported, but should be interpreted with caution because most composites scores had non-normal distributions. A Cohen's *d* of 0.2 is considered small, 0.5 medium, and 0.8 large (36). The effect sizes are coded so that a positive effect size indicates better performance at the 3-month assessment. It is important to note that these effect size interpretation criteria are guidelines, and that whether an effect of a certain size is important or not depends on the context (e.g., in the present study, the effect sizes of different composites should be compared against each other, rather than against Cohen's benchmarks) (38). Spearman's *rho* was used to examine test-retest reliability for the composite scores between the 2-week assessment and the 3-month assessment. Because most composite scores were, by design, zero-inflated and non-normally distributed, reliable change was calculated from the natural distribution of the difference scores. First, the difference scores were calculated by subtracting the 2-week score from the 3-month score. The natural distributions were then examined to identify “uncommon” and “very uncommon” difference scores. Those correspond to improvements or declines in performance that are experienced by 20% or fewer or 10% or fewer of each sample (i.e., the 10, 20, 80, and 90th percentiles of the distribution of difference scores). The percentiles were identified with the default HAVARAGE procedure in IBM SPSS Statistics v.25. Of note, when using the HAVERAGE method in contexts where the exact percentile of interest in the natural distribution does not exist (e.g., no score would correspond exactly to the 10th percentile in a sample of 63 participants, such as the community control group), then the score is interpolated from scores surrounding the percentile of interest.

## Results

### Participant Characteristics

There were 140 adults with MTBIs who completed the 2-week assessment and 124 of them (88.6%) completed the 3-month assessment. The MTBI sample (*n* = 124; 27.4% women) was an average age of 33.4 years old (Mdn = 30.4, SD = 12.3, range = 18.1–59.7), with an average of 14.3 years of education (Mdn = 13.0, SD = 2.5, range = 10.0–21.0). There were 72 adults in the trauma control sample who completed the 2-week assessment and 67 of them (93.1%) completed the 3-month assessment. The trauma control sample (*n* = 67; 38.8% women) was an average age of 32.3 years old (median = 27.5, SD = 12.7, range = 18.1–59.8), with an average of 14.7 years of education (Mdn = 15.0, SD = 2.6, range = 10.0–21.0). There were 70 adults in the community control sample who completed the 2-week assessment and 63 of them (90.0%) completed the 3-month assessment. The community control sample (*n* = 63; 39.7% women) was an average age of 34.1 years old (Mdn = 29.9, SD = 12.3, range = 18.7–58.6) with an average of 14.3 years of education (median = 14.0, SD = 2.3, range = 10.0–18.0). There were no significant differences in age (*p* = 0.464), years of education (*p* = 0.710), or gender representation (*p* = 0.136) between groups. The most common cause of MTBI was a fall (*n* = 48, 38.7%), and the majority of the MTBI group were discharged (i.e., not admitted to any other department, such as the neurosurgery department) from the emergency clinics (*n* = 90, 72.6%). Traumatic intracranial findings were found in 16 (12.9%) of the patients with MTBI, 87 (70.2%) had PTA present for <1 h, and 37 (29.8%) had PTA for 1–24 h.

### Descriptive Statistics for the Scores and Composite Scores

Scores from the individual tests that constitute the composite scores are reported in Table 1. For all groups, all test scores appeared higher on the 3-month assessment than on the 2-week assessment, with the exception of the RAVLT delayed trial. The descriptive characteristics of the composite scores at 2 weeks and 3 months are shown in Table 2 for the MTBI group and in Table 3 for the control groups. All of the combined battery composite scores, except the # ≤ 5th %tile, identified significantly better cognitive performances at the 3-month assessment compared to the 2-week assessment in all groups (Tables 2, 3). Differences, in terms of effect sizes, were largest on the OTBM composites (for the combined battery: MTBI: *r* = 0.42; trauma controls: *r* = 0.43; community controls: *r* = 0.41).

**Table 1:** Descriptive statistics of the individual test scores.

	**Two weeks**			**Three months**		
	**Mild traumatic brain injury**	**Trauma controls**	**Community controls**	**Mild traumatic brain injury**	**Trauma controls**	**Community controls**
**Trail making test part A**						
Raw, *M, Mdn*	26.12, 25.00	24.81, 25.00	25.27, 23.00	22.65, 22.00	21.52, 21.00	23.29, 22.00
Raw, *SD*	8.57	7.34	8.88	6.45	5.21	7.08
Raw, Interquartile range	20.00–30.00	19.00–28.00	19.00–30.00	18.00–27.00	17.00–26.00	18.00–28.00
T score, *M, Mdn*	50.08, 51.52	51.46, 52.69	51.53, 54.00	54.14, 54.93	55.33, 56.40	54.00, 53.57
T score, *SD*	10.27	8.64	9.10	8.13	6.26	6.68
T score, Interquartile range	44.96–57.26	46.13–57.82	44.07–58.64	49.86–59.83	49.61–60.45	49.40–58.30
**Trail making test part B**						
Raw, *M, Mdn*	64.38, 58.50	62.73, 55.00	61.54, 55.00	57.76, 53.00	53.67, 47.00	56.60, 51.00
Raw, *SD*	27.32	28.50	24.13	26.04	21.16	31.52
Raw, Interquartile range	44.25–73.75	46.00–68.00	44.00–75.00	43.00–66.75	41.00–64.00	40.00–65.00
T score, *M, Mdn*	48.14, 51.02	49.48, 52.45	49.86, 51.47	51.52, 54.37	52.97, 54.93	52.69, 54.51
T score, *SD*	12.34	9.54	9.54	10.35	9.65	9.65
T score, Interquartile range	43.24–57.25	44.49–55.61	43.71–57.80	47.33–57.71	48.82–59.90	48.97–58.86
**RAVLT-Trial 1 to 5**						
Raw, *M, Mdn*	50.46, 52.00	53.28, 54.00	52.81, 54.00	51.87, 52.50	53.63, 54.00	53.76, 54.00
Raw, *SD*	8.96	8.82	8.87	9.78	8.65	8.55
Raw, Interquartile range	44.00–56.00	47.00–60.00	45.00–60.00	44.00–59.75	47.00–59.00	47.00–60.00
T score, *M, Mdn*	45.79, 47.56	49.37, 49.88	49.05, 48.02	47.66, 48.35	49.92, 50.41	50.31, 49.88
T score, *SD*	11.46	10.61	10.83	12.20	9.98	10.00
T score, Interquartile range	38.69–53.15	42.19–57.61	40.62–58.63	38.30–56.41	42.69–57.71	43.56–56.86
**RAVLT-Delayed recall**						
Raw, *M, Mdn*	10.60, 11.00	11.66, 12.00	11.13, 12.00	10.44, 11.00	11.10, 12.00	11.30, 11.00
Raw, *SD*	2.93	2.61	2.87	3.14	2.79	2.71
Raw, Interquartile range	9.00–13.00	10.00–14.00	9.00–13.00	8.00–13.00	9.00–13.00	10.00–14.00
T score, *M, Mdn*	49.01, 49.64	52.60, 53.21	50.93, 52.80	48.50, 49.22	50.56, 52.80	51.69, 52.80
T score, *SD*	10.50	9.49	10.51	11.09	9.88	9.52
T score, Interquartile range	42.89–56.80	45.71–60.80	44.06–56.80	38.57–56.80	42.27–56.79	44.80–60.36
**CANTAB-Paired associates learning**						
Raw, *M, Mdn*	8.44, 6.00	7.58, 5.00	8.76, 6.00	6.49, 4.00	6.84, 4.00	6.00, 4.00
Raw, *SD*	8.17	7.62	9.07	6.75	7.97	6.98
Raw, Interquartile range	3.00–11.00	2.00–10.00	3.00–12.00	2.00–9.00	2.00–9.00	1.00–8.00
T score, *M, Mdn*	51.26, 52.66	51.74, 53.97	51.95, 53.77	53.10, 54.04	52.78, 53.97	54.20, 55.02
T score, *SD*	6.55	5.96	6.38	5.99	5.43	4.98
T score, Interquartile range	47.46–55.96	49.39–55.96	48.75–56.58	51.21–56.90	50.33–56.05	52.21–58.37
**CANTAB-Rapid visual processing**						
Raw, *M, Mdn*	0.91, 0.91	0.92, 0.92	0.91, 0.92	0.93, 0.94	0.94, 0.95	0.93, 0.94
Raw, *SD*	0.05	0.05	0.05	0.05	0.04	0.05
Raw, Interquartile range	0.88–0.95	0.90–0.95	0.88–0.94	0.90–0.98	0.91–0.98	0.91–0.95
T score, *M, Mdn*	47.58, 48.63	49.87, 50.23	47.68, 50.52	52.63, 53.80	54.59, 57.93	51.48, 53.80
T score, *SD*	11.80	10.45	9.93	11.53	9.88	11.33
T score, Interquartile range	39.85–56.57	43.78–56.74	41.93–54.60	46.37–62.30	46.37–62.60	45.78–58.47
**CANTAB-Spatial working memory**						
Raw, *M, Mdn*	13.33, 11.00	14.79, 11.00	16.52, 11.00	11.53, 8.50	12.66, 9.00	13.68, 10.00
Raw, *SD*	12.97	15.56	16.74	12.83	14.87	14.73
Raw, Interquartile range	2.00–22.00	2.00–24.00	3.00–29.00	1.00–16.75	1.00–19.00	2.00–22.00
T score, *M, Mdn*	53.63, 55.28	52.03, 54.39	52.72, 53.82	54.96, 56.79	53.46, 56.87	54.44, 56.19
T score, *SD*	7.80	9.69	8.05	7.09	9.55	6.57
T score, Interquartile range	49.11–59.72	44.63–60.34	49.08–58.83	50.87–60.34	49.96–59.72	50.86–59.72
**CANTAB-Reaction time index**						
Raw, *M, Mdn*	334.8, 331.9	328.8, 328.4	321.3, 315.6	331.8, 328.8	325.8, 318.4	320.7, 316.6
Raw, *SD*	45.2	41.8	42.1	41.6	46.0	42.0
Raw, Interquartile range	300.5–352.3	298.5–351.5	295.4–346.4	302.5–352.5	287.0–363.8	295.0–338.6
T score, *M, Mdn*	52.59, 53.46	53.59, 53.19	55.56, 56.54	53.25, 53.93	54.27, 55.20	55.62, 57.01
T score, *SD*	8.71	8.25	7.91	8.19	9.05	7.98
T score, Interquartile range	48.45–59.26	48.10–58.68	50.93–60.79	49.62–58.36	48.04–60.75	52.16–61.00
**CANTAB-Stockings of Cambridge**						
Raw, *M, Mdn*	9.75, 10.00	9.54, 10.00	9.43, 10.00	10.18, 10.50	10.22, 11.00	9.89, 10.00
Raw, *SD*	1.72	1.99	2.01	1.65	1.52	1.59
Raw, Interquartile range	8.25–11.00	8.00–11.00	8.00–11.00	9.00–12.00	9.00–11.00	9.00–11.00
T score, *M, Mdn*	53.27, 53.68	51.93, 52.87	51.92, 54.23	55.60, 58.86	55.62, 58.86	54.39, 54.23
T score, *SD*	9.08	10.20	10.00	8.67	8.27	7.70
T score, Interquartile range	46.89–59.40	44.85–59.42	44.85–59.38	49.07–64.27	49.07–59.42	49.07–59.38

*Sample sizes are as follows: Mild Traumatic Brain Injury group = 124, trauma controls = 67, and community controls = 63; RAVLT = Rey Auditory Verbal Learning Test*.

**Table 2:** Within group comparisons (2-week and 3-month assessments), *p*-values, and effect sizes (*r*, Cohen's *d*) in the mild traumatic brain injury group.

	**Two weeks**		**Three months**			
**Composites**	***M, Mdn***	***SD, IQR***	***M, Mdn***	***SD, IQR***	***p***	***r, d***
**Paper-and-pencil (4 scores)**						
OTBM	48.26, 49.18	8.70, 43.82–54.67	50.46, 51.31	7.84, 44.70–57.17	<0.001	0.24, 0.27
GDS	0.46, 0.00	0.83, 0.00–0.50	0.36, 0.00	0.56, 0.00–0.50	0.239	0.07, 0.14
NDS-W	0.87, 0.38	1.19, 0.06–1.19	0.69, 0.25	0.88, 0.00–1.09	0.052	0.12, 0.17
LSC	44.81, 47.08	6.32, 42.55–49.46	45.85, 47.98	4.75, 42.59–50.00	0.027	0.14, 0.19
# ≤ 5th %tile	0.48, 0.00	0.87, 0.00–1.00	0.40, 0.00	0.73, 0.00–1.00	0.260	0.07, 0.10
# ≤ 16th %tile	0.92, 0.00	1.17, 0.00–2.00	0.81, 0.00	1.02, 0.00–2.00	0.319	0.06, 0.10
# <50th %tile	1.99, 2.00	1.42, 1.00–3.00	1.65, 2.00	1.34, 0.00–3.00	0.003	0.19, 0.25
**CANTAB (5 scores)**						
OTBM	51.67, 52.03	5.35, 49.07–56.06	53.91, 54.63	5.22, 50.22–57.67	<0.001	0.40, 0.42
GDS	0.21, 0.00	0.38, 0.00–0.20	0.13, 0.00	0.28, 0.00–0.00	0.006	0.18, 0.24
NDS-W	0.49, 0.25	0.61, 0.06–0.65	0.31, 0.10	0.46, 0.00–0.40	<0.001	0.27, 0.34
LSC	47.11, 48.21	3.18, 46.15–49.48	48.10, 49.15	2.63, 47.02–50.00	<0.001	0.32, 0.34
# ≤ 5th %tile	0.29, 0.00	0.57, 0.00–0.00	0.21, 0.00	0.51, 0.00–0.00	0.139	0.09, 0.15
# ≤ 16th %tile	0.60, 0.00	0.83, 0.00–1.00	0.37, 0.00	0.72, 0.00–1.00	0.001	0.21, 0.30
# <50th %tile	1.86, 2.00	1.20, 1.00–3.00	1.39, 1.00	1.28, 0.00–2.00	<0.001	0.28, 0.38
**Combined (9 scores)**						
OTBM	50.15, 51.30	5.98, 46.20–54.43	52.38, 53.07	5.66, 47.92–56.39	<0.001	0.42, 0.38
GDS	0.32, 0.11	0.49, 0.00–0.33	0.23, 0.11	0.35, 0.00–0.33	0.004	0.18, 0.21
NDS-W	0.66, 0.40	0.74, 0.14–0.92	0.48, 0.31	0.57, 0.06–0.69	<0.001	0.27, 0.27
LSC	46.09, 47.45	3.94, 44.41–49.01	47.10, 48.01	3.13, 45.73–49.52	<0.001	0.28, 0.29
# ≤ 5th %tile	0.77, 0.00	1.18, 0.00–1.00	0.61, 0.00	1.07, 0.00–1.00	0.084	0.11, 0.15
# ≤ 16th %tile	1.52, 1.00	1.62, 0.00–2.75	1.19, 1.00	1.39, 0.00–2.00	0.006	0.18, 0.22
# <50th %tile	3.85, 4.00	2.20, 2.00–5.75	3.04, 3.00	2.15, 1.00–5.00	<0.001	0.33, 0.37

*Sample size = 124. #, Number of scores; d, Cohen's d; GDS, Global Deficit Score; LSC, Low Score Composite; NDS-W, Neuropsychological Deficit Score-Weighted; OTBM, Overall Test Battery Mean. P-values are from Wilcoxon Signed-Rank Test. A positive effect size indicates better performance at the 3-month assessment. An r of 0.1 is considered a small effect size, 0.3 a medium effect size, and 0.5 a large effect size. Cohen's ds should be interpreted with caution because of the non-normal distribution characterizing most of the composite scores. A Cohen's d of 0.2 is considered small, 0.5 medium, and 0.8 large*.

**Table 3:** Within group comparisons (2-week and 3-month assessments), *p*-values, and effect sizes (*r*, Cohen's *d*) in the control groups.

	**Trauma control group (*****n*** **=** **67)**						**Community control group (*****n*** **=** **63)**					
	**Two weeks**		**Three months**				**Two weeks**		**Three months**			
**Composites**	***M, Mdn***	***SD, IQR***	***M, Mdn***	***SD, IQR*,**	***p***	***r, d***	***M, Mdn***	***SD, IQR***	***M, Mdn***	***SD, IQR***	***p***	***r, d***
**Paper-and-pencil (4 scores)**												
OTBM	50.73, 51.78	7.02, 46.49–55.81	52.20, 53.66	6.72, 47.60–57.22	0.018	0.20, 0.21	50.34, 50.85	7.30, 43.97–55.50	52.17, 52.54	6.17, 48.54–56.76	0.013	0.22, 0.27
GDS	0.25, 0.00	0.44, 0.00–0.25	0.19, 0.00	0.47, 0.00–0.25	0.209	0.11, 0.13	0.30, 0.00	0.45, 0.00–0.50	0.15, 0.00	0.38, 0.00–0.00	0.024	0.20, 0.36
NDS-W	0.54, 0.25	0.74, 0.06–0.81	0.42, 0.13	0.69, 0.00–0.63	0.161	0.12, 0.17	0.63, 0.31	0.74, 0.06–1.00	0.42, 0.13	0.59, 0.06–0.63	0.023	0.20, 0.32
LSC	46.53, 48.21	4.29, 45.23–49.91	47.26, 48.81	4.03, 45.37–50.00	0.073	0.15, 0.18	46.05, 47.47	4.20, 43.37–49.82	47.42, 49.08	3.40, 46.09–49.83	0.010	0.23, 0.36
# ≤ 5th %tile	0.25, 0.00	0.59, 0.00–0.00	0.21, 0.00	0.64, 0.00–0.00	0.603	0.04, 0.07	0.33, 0.00	0.62, 0.00–1.00	0.16, 0.00	0.45, 0.00–0.00	0.052	0.17, 0.32
# ≤ 16th %tile	0.64, 0.00	0.95, 0.00–1.00	0.51, 0.00	0.84, 0.00–1.00	0.229	0.10, 0.15	0.84, 0.00	1.03, 0.00–2.00	0.44, 0.00	0.74, 0.00–1.00	0.005	0.25, 0.45
# <50th %tile	1.70, 2.00	1.31, 1.00–2.00	1.34, 1.00	1.27, 0.00–2.00	0.016	0.21, 0.28	1.71, 2.00	1.38, 1.00–3.00	1.59, 2.00	1.17, 1.00–2.00	0.402	0.07, 0.09
**CANTAB (5 scores)**												
OTBM	51.83, 51.72	5.98, 48.24–56.89	54.14, 55.01	5.60, 50.69–57.72	<0.001	0.42, 0.40	51.97, 52.48	5.27, 48.85–55.02	54.03, 54.39	4.64, 51.03–57.02	<0.001	0.35, 0.42
GDS	0.19, 0.00	0.35, 0.00–0.40	0.12, 0.00	0.30, 0.00–0.00	0.016	0.21, 0.22	0.20, 0.00	0.33, 0.00–0.20	0.12, 0.00	0.24, 0.00–0.20	0.023	0.20, 0.24
NDS-W	0.46, 0.30	0.56, 0.05–0.65	0.31, 0.10	0.51, 0.00–0.40	<0.001	0.30, 0.28	0.43, 0.25	0.54, 0.05–0.55	0.27, 0.10	0.40, 0.00–0.35	0.001	0.28, 0.34
LSC	47.19, 48.04	3.16, 46.25–49.74	48.08, 49.02	2.90, 47.41–50.00	<0.001	0.30, 0.29	47.34, 48.45	3.11, 46.59–49.99	48.27, 49.23	2.50, 47.11–50.00	0.002	0.28, 0.33
# ≤ 5th %tile	0.28, 0.00	0.57, 0.00–0.00	0.19, 0.00	0.58, 0.00–0.00	0.180	0.12, 0.16	0.27, 0.00	0.54, 0.00–0.00	0.16, 0.00	0.37, 0.00–0.00	0.035	0.19, 0.24
# ≤ 16th %tile	0.67, 0.00	1.01, 0.00–1.00	0.40, 0.00	0.76, 0.00–1.00	0.003	0.26, 0.31	0.59, 0.00	0.87, 0.00–1.00	0.38, 0.00	0.68, 0.00–1.00	0.026	0.20, 0.27
# <50th %tile	1.88, 2.00	1.39, 1.00–3.00	1.36, 1.00	1.31, 0.00–2.00	<0.001	0.33, 0.39	1.67. 2.00	1.36, 1.00–3.00	1.16, 1.00	1.08, 0.00–2.00	0.002	0.27, 0.42
**Combined (9 scores)**												
OTBM	51.34, 52.16	5.56, 49.05–55.08	53.28, 53.74	5.13, 50.64–57.36	<0.001	0.43, 0.36	51.24, 51.32	5.05, 47.80–55.30	53.20, 53.27	4.42, 51.06–56.63	<0.001	0.41, 0.41
GDS	0.22, 0.11	0.33, 0.00–0.22	0.15, 0.00	0.27, 0.00–0.22	0.017	0.21, 0.23	0.24, 0.11	0.29, 0.00–0.44	0.14, 0.00	0.23, 0.00–0.22	0.001	0.29, 0.38
NDS-W	0.49, 0.28	0.56, 0.14–0.67	0.36, 0.19	0.44, 0.06–0.44	0.002	0.27, 0.26	0.52, 0.39	0.50, 0.11–0.78	0.33, 0.17	0.38, 0.06–0.50	<0.001	0.32, 0.43
LSC	46.90, 48.00	3.22, 45.61–49.10	47.72, 48.59	2.63, 46.77–49.59	0.001	0.28, 0.28	46.77, 47.49	2.90, 45.13–49.22	47.89, 48.74	2.31, 47.06–49.72	<0.001	0.32, 0.43
# ≤ 5th %tile	0.54, 0.00	0.96, 0.00–1.00	0.40, 0.00	0.85, 0.00–0.00	0.214	0.11, 0.15	0.60, 0.00	0.85, 0.00–1.00	0.32, 0.00	0.62, 0.00–1.00	0.012	0.22, 0.38
# ≤ 16th %tile	1.31, 1.00	1.63, 0.00–2.00	0.91, 0.00	1.23, 0.00–2.00	0.006	0.24, 0.28	1.43, 1.00	1.42, 0.00–2.00	0.83, 0.00	1.12, 0.00–1.00	<0.001	0.32, 0.47
# <50th %tile	3.58, 3.00	2.26, 2.00–5.00	2.70, 2.00	2.19, 1.00–4.00	<0.001	0.37, 0.40	3.38, 3.00	2.25, 1.00–5.00	2.75, 3.00	1.79, 1.00–4.00	0.002	0.28, 0.31

*#, Number of scores; d, Cohen's d; GDS, Global Deficit Score; LSC, Low Score Composite; NDS-W, Neuropsychological Deficit Score-Weighted; OTBM, Overall Test Battery Mean. P-values are from Wilcoxon Signed-Rank Test. A positive effect size indicates better performance at the 3-month assessment. An r of 0.1 is considered a small effect size, 0.3 a medium effect size, and 0.5 a large effect size. Cohen's ds should be interpreted with caution because of the non-normal distribution characterizing most of the composite scores. A Cohen's d of 0.2 is considered small, 0.5 medium, and 0.8 large*.

The percentage of participants who had at least 1, 2, or 3 low scores on the test batteries (i.e., base rates of low scores) are shown in Table 4. Having at least one score at or below the 5th percentile was common in all three groups and especially when both the paper-and-pencil tests and the CANTAB tests were included (i.e., the combined battery). For the 2-week assessment, the percentage of participants with one or more scores at or below the 5th percentile was 41.1% for the MTBI group, 32.8% for the trauma control group, and 39.7% for the community control group. In general, the base rates of low scores were lower on the 3-month assessment compared to the 2-week assessment, and when the paper-and-pencil and CANTAB batteries were examined separately.

**Table 4:** The percentages of the participants who have *at least* 1, 2, or 3 low scores, by group and time point.

	**Mild traumatic brain injury (*****n*** **=** **124)**						**Trauma controls (*****n*** **=** **67)**						**Community controls (*****n*** **=** **63)**					
	**Number of low scores Two weeks**			**Number of low scores Three months**			**Number of low scores Two weeks**			**Number of low scores Three months**			**Number of low scores Two weeks**			**Number of low scores Three months**		
**Composites**	**1+**	**2+**	**3+**	**1+**	**2+**	**3+**	**1+**	**2+**	**3+**	**1+**	**2+**	**3+**	**1+**	**2+**	**3+**	**1+**	**2+**	**3+**
**Paper-and-pencil (4 scores)**																		
# ≤ 5th %tile	29.0	14.5	4.0	25.8	12.9	0.8	17.9	7.5	0.0	11.9	6.0	3.0	25.4	7.9	0.0	12.7	3.2	0.0
# ≤ 16th %tile	48.4	27.4	11.3	46.8	27.4	5.6	40.3	14.9	9.0	32.8	13.4	4.5	47.6	27.0	9.5	31.7	11.1	1.6
# <50th %tile	81.5	58.1	38.7	74.2	52.4	25.8	79.1	52.2	23.9	64.2	43.3	20.9	76.2	50.8	28.6	77.8	52.4	23.8
**CANTAB (5 scores)**																		
# ≤ 5th %tile	23.4	5.6	0.0	16.9	3.2	0.8	22.4	6.0	0.0	11.9	6.0	1.5	22.2	4.8	0.0	15.9	0.0	0.0
# ≤ 16th %tile	40.4	16.9	2.4	26.6	8.1	1.6	40.3	16.4	9.0	26.9	10.4	3.0	38.1	15.9	4.8	28.6	7.9	1.6
# <50th %tile	87.1	58.1	31.5	68.5	41.9	19.4	79.1	58.2	35.8	70.1	35.8	19.4	76.2	50.8	25.4	66.7	34.9	11.1
**Combined (9 scores)**																		
# ≤ 5th %tile	41.1	19.4	11.3	33.1	16.9	5.6	32.8	11.9	6.0	22.4	11.9	6.0	39.7	17.5	3.2	25.4	4.8	1.6
# ≤ 16th %tile	62.1	42.7	25.0	57.3	33.1	16.1	58.2	28.4	22.4	46.3	25.4	13.4	65.1	42.9	22.2	46.0	22.2	11.1
# <50th %tile	96.8	82.3	68.5	89.5	71.0	53.2	91.0	85.1	67.2	82.1	64.2	49.3	90.5	74.6	60.3	92.1	71.4	54.0

*#, Number of scores. The table shows the percentages of the participants who has at least 1, 2, or 3 low scores, by group and time point*.

### Test-Retest Reliability and Reliable Change

The test-retest correlations were higher in the combined composites than in the paper-and-pencil and CANTAB composites (Table 5). The OTBM composite had the highest test-retest correlation (i.e., 0.87 for the combined composite in the community control group). The lowest test-retest correlations in the community control group were observed for the paper-and-pencil #≤5th %tile (0.14) and GDS (0.28).

**Table 5:** Test-retest correlations, Spearman's *rho*.

	**MTBI**	**Trauma controls**	**Community controls**
**Paper-and-pencil (4 scores)**			
OTBM	0.74**	0.64**	0.70**
GDS	0.52**	0.49**	0.28*
NDS-W	0.61**	0.53**	0.59**
LSC	0.61**	0.53**	0.57**
# ≤ 5th %tile	0.54**	0.10	0.14
# ≤ 16th %tile	0.53**	0.41**	0.39**
# <50th %tile	0.59**	0.52**	0.66**
**CANTAB (5 scores)**			
OTBM	0.82**	0.82**	0.71**
GDS	0.53**	0.47**	0.55**
NDS-W	0.65**	0.70**	0.58**
LSC	0.67**	0.69**	0.59**
# ≤ 5th %tile	0.34**	0.41**	0.63**
# ≤ 16th %tile	0.55**	0.63**	0.48**
# <50th %tile	0.60**	0.71**	0.53**
**Combined (9 scores)**			
OTBM	0.85**	0.83**	0.87**
GDS	0.60**	0.48**	0.55**
NDS-W	0.72**	0.65**	0.68**
LSC	0.73**	0.66**	0.67**
# ≤ 5th %tile	0.53**	0.28*	0.41**
# ≤ 16th %tile	0.58**	0.56**	0.61**
# <50th %tile	0.73**	0.68**	0.76**
**Individual tests**			
TMT Part A	0.60**	0.49**	0.42**
TMT Part B	0.74**	0.67**	0.66**
RAVLT trial 1 to 5	0.58**	0.59**	0.56**
RAVLT delayed	0.51**	0.60**	0.55**
Paired associates learning	0.43**	0.57**	0.40**
Rapid visual processing	0.73**	0.66**	0.65**
Spatial working memory	0.62**	0.64**	0.61**
Reaction time index	0.62**	0.75**	0.68**
Stockings of Cambridge	0.52**	0.53**	0.33**

Sample sizes are as follows: MTBI group = 124, trauma controls = 67, and community controls = 63. #, Number of scores; GDS, Global Deficit Score; LSC, Low Score Composite; MTBI, Mild Traumatic Brain Injury; NDS-W, Neuropsychological Deficit Score-Weighted; OTBM, Overall Test Battery Mean; RAVLT, Rey Auditory Verbal Learning Test; TMT, Trail Making Test. **p < 0.01; *p < 0.05.

Reliable changes on each composite from 2 weeks to 3 months based on the natural distribution of composite change scores are shown in Table 6. As an example, the cutoff value for improvement at the 90th percentile (i.e., being among the 10% with greatest improvement) for the combined OTBM composite in the community control group was +5.94, which means if an individual's change on the OTBM from 2 weeks to 3 months exceeds +5.94, that individual would have shown greater improvement than 90% of the community control group. Notably, if the exact percentile of interest in the natural distribution of difference scores does not exist (e.g., no score correspond exactly to the 90th percentile in a sample of 63 participants, such as the community control group), then the score is interpolated from the scores surrounding the percentile of interest (i.e., the default HAVERAGE procedure for calculating percentiles in SPSS). Consequently, some of the scores in Table 6 do not exist in the natural distribution of the difference score, and some are even theoretically impossible scores for an individual participant (e.g., 0.6, 10th percentile, #≤16th %tile paper-and-pencil composite, community control group).

**Table 6:** Interpreting change on the composite scores based on the natural distribution of difference scores.

	**MTBI group (*n* = 124)**				**Trauma control group (*n* = 67)**				**Community control group (*n* = 63)**			
	**Decline**		**Improvement**		**Decline**		**Improvement**		**Decline**		**Improvement**	
	**Very uncommon**	**Uncommon**	**Uncommon**	**Very uncommon**	**Very uncommon**	**Uncommon**	**Uncommon**	**Very uncommon**	**Very uncommon**	**Uncommon**	**Uncommon**	**Very uncommon**
	**≤10%**	**≤20%**	**≤20%**	**≤10%**	**≤10%**	**≤20%**	**≤20%**	**≤10%**	**≤10%**	**≤20%**	**≤20%**	**≤10%**
**Paper-and-pencil (4 scores)**												
OTBM	−5.14	−2.96	+6.57	+9.77	−6.24	−3.09	+5.77	+8.43	−5.66	−3.37	+5.58	+8.79
GDS	+0.50	+0.25	−0.25	−0.75	+0.50	+0.00	−0.25	−0.50	+0.40	+0.00	−0.50	−1.00
NDS-W	+0.72	+0.31	−0.50	−1.16	+0.51	+0.28	−0.53	−0.85	+0.66	+0.13	−0.65	−1.40
LSC	−4.92	−1.96	+3.37	+7.04	−3.50	−1.77	+3.20	+5.12	−3.11	−0.93	+4.02	+7.41
# ≤ 5th %tile	+1.00	+0.00	−0.00	−1.00	+1.00	+0.00	−0.00	−1.00	+0.00	+0.00	−1.00	−1.00
# ≤ 16th %tile	+1.00	+1.00	−1.00	−1.50	+1.00	+0.00	−1.00	−1.00	+0.60	+0.00	−1.00	−2.00
# <50th %tile	+1.00	+1.00	−1.00	−2.00	+1.00	+1.00	−1.40	−2.00	+1.00	+1.00	−1.00	−1.60
**CANTAB (5 scores)**												
OTBM	−2.71	−0.38	+4.84	+5.91	−2.09	−0.35	+4.81	+5.80	−2.07	−1.24	+5.54	+6.73
GDS	+0.20	+0.00	−0.20	−0.40	+0.04	+0.00	−0.20	−0.40	+0.20	+0.00	−0.20	−0.40
NDS-W	+0.23	+0.10	−0.40	−0.75	+0.25	+0.00	−0.40	−0.50	+0.23	+0.06	−0.35	−0.63
LSC	−1.37	−0.20	+2.40	+3.70	−1.46	−0.30	+2.19	+3.09	−1.72	−0.60	+2.45	+3.58
# ≤ 5th %tile	+1.00	+0.00	−0.00	−1.00	+0.20	+0.00	−0.00	−1.00	+0.00	+0.00	−0.00	−1.00
# ≤ 16th %tile	+0.00	+0.00	−1.00	−1.00	+0.00	+0.00	−1.00	−1.00	+1.00	+0.00	−1.00	−1.00
# <50th %tile	+1.00	+1.00	−1.00	−2.00	+1.00	+0.00	−1.00	−2.00	+1.00	+0.00	−1.00	−2.00
**Combined (9 score)**												
OTBM	−1.57	−0.44	+4.65	+6.25	−1.75	−0.51	+3.98	+5.90	−2.33	−0.38	+4.17	+5.94
GDS	+0.22	+0.11	−0.22	−0.44	+0.22	+0.00	−0.22	−0.44	+0.11	+0.00	−0.22	−0.56
NDS-W	+0.22	+0.11	−0.44	−0.69	+0.23	+0.09	−0.39	−0.52	+0.21	+0.08	−0.48	−0.77
LSC	−1.40	−0.82	+2.61	+3.72	−1.18	−0.59	+2.46	+3.14	−1.53	−0.49	+2.74	+4.32
# ≤ 5th %tile	+1.00	+0.00	−1.00	−1.50	+1.00	+0.00	−1.00	−1.00	+0.60	+0.00	−1.00	−1.00
# ≤ 16th %tile	+1.00	+0.00	−1.00	−2.00	+1.00	+0.00	−1.00	−2.00	+1.00	+0.00	−1.20	−2.00
# <50th %tile	+1.00	+1.00	−2.00	−3.00	+1.00	+0.00	−2.00	−3.00	+1.00	+1.00	−2.00	−3.00

*#, Number of scores; GDS, Global Deficit Score; LSC, Low Score Composite; NDS-W, Neuropsychological Deficit Score-Weighted; OTBM, Overall Test Battery Mean. The percentiles are calculated with the HAVARAGE method in SPSS. The natural distributions were examined to identify “uncommon” and “very uncommon” difference scores. Those correspond to improvements or declines in performance that are experienced by 20% or fewer or 10% or fewer of each sample (i.e., the 10, 20, 80, and 90th percentiles of the distribution of difference scores). If the exact percentile asked for in the natural distribution of difference scores does not exist (i.e., no score correspond exactly to the 10th percentile in a sample of 63 participants, such as the community control group), then the score is interpolated from the scores surrounding the percentile asked for. The + and – signs indicate that a change needs to be greater or less than the specified amount, respectively, to be considered reliable change. When the cutoff score for change is 0, that means that a deviation from 0 indicates a reliable change (e.g., having one more or one fewer low scores, at or below the 5th percentile, on retesting, is considered to be a reliable change for the CANTAB battery)*.

## Discussion

The present study examined longitudinally seven candidate composite scores for the neuropsychological test battery used in CENTER-TBI. Mean normative scores for the individual tests were mostly in the normal range at 2 weeks in all groups with modestly higher scores at 3 months in all three groups (Table 1). There was some variability in mean normative scores, with RAVLT Trials 1–5 being lower and some CANTAB scores being higher (e.g., Spatial Working Memory). There were small statistically significant improvements on nearly all of the composites from 2 weeks to 3 months across groups (Tables 2, 3). Somewhat larger improvements were seen on the OTBM composite scores, suggesting this score may be more sensitive to change in cognitive performance. The OTBM composite more directly aggregates improvements across the individual test scores and this may explain why improvement was larger on this composite. However, with all groups improving in similar magnitude, change from 2 weeks to 3 months likely reflects a common cause across groups (i.e., practice) whereas if the MTBI group improved to a greater degree, that change would have likely corresponded to cognitive recovery following injury. Thus, the OBTM might be the most sensitive composite for detecting practice effects, but not necessarily for detecting cognitive recovery.

It is important to note that low test scores were common in this study across all groups. A considerable portion of the trauma control group (32.8%) and the community control group (39.7%) had at least one individual test score at or below the 5th percentile at the 2-week assessment, when all nine scores were considered. Further, the portion of the control groups that had 3+ (out of 9 total) scores at or below the 16th percentile was about 22% at the 2-week assessment and 11–13% at the 3-month assessment. This finding aligns with previous studies on multivariate base rates, that have consistently demonstrated that low scores occur commonly among cognitively healthy individuals (26–33, 39). As prior studies have noted, it is essential to consider the base rates of low scores in control/normative samples when interpreting low scores in clinical samples.

The OTBM composite had the highest test-retest reliability (Table 5). There is no generally accepted cutoff for what constitutes adequate test-retest reliability (14). Using the guidelines from Strauss et al. (18) for individual tests, a test-retest correlation <0.60 is low, 0.60–0.69 is marginal, 0.70–0.79 is adequate, and ≥0.80 is high. With these reference values, the OTBM composite had a high test-retest correlation (*rho* = 0.87, for the combined composite in the community control group) and the #<50th %tile composite had adequate reliability (*rho* = 0.76, for the combined composite in the community control group). The test-retest correlations for the other composites fell in the low or marginal category. A test-retest correlation of 0.87 is considerable higher than the test-retest correlations for the individual tests in the present study (Table 5) and for most of the tests in the CANTAB battery (40–43). The test-retest reliability was similar for the paper-and-pencil composite (e.g., OTBM *rho* = 0.70 in the community control group) and the CANTAB composite (e.g., OTBM *rho* = 0.71 in the community control group) and these particular results suggest that the CANTAB tests are not inferior or superior to the traditional paper-and-pencil tests. It is notable, although expected, that test-retest reliability increases in association with greater numbers of tests included in the composite scores. Even if the reliability is inadequate for many of the individual tests, the reliability is adequate for the paper-and-pencil composite and the CANTAB composite and high for the combined composite. This favors the use of composite scores in research and clinical trials and can, to some extent, compensate for low test-retest reliability observed for many individual neuropsychological tests. Further, the cognitive domains most affected by MTBI show great variability between studies (44), suggesting between-patient variability in cognitive deficits (e.g., some patients present with mainly attentional deficits and others with memory deficits). Under these circumstances, a cognitive composite score that sums deficit scores might be better suited than individual tests for detecting cognitive deficits in MTBI research and clinical trials.

Using deficit-based scores (i.e., all the composites except the OTBM) in longitudinal studies is complicated by practice effects, which are expected on neuropsychological tests (41, 45). Even in the absence of cognitive recovery, fewer participants are expected to fulfill a criterion for defining a cognitive deficit (e.g., having two or more scores at or below the 5th percentile) on a second assessment because of practice effects. An individual who obtains a low score on the first assessment (e.g., ≤5th percentile) and benefited from a practice effect may still obtain a low score, but this score may now exceed the threshold that would be quantified as a low score (e.g., on retest, the score falls at the 9th percentile). Because normative data does not typically consider practice effects (i.e., the normative sample has not been exposed to repeated neuropsychological testing), this is an inherent problem with using deficit-based composite scores that are based on normative data. In comparison to deficit scores, the test-retest correlation for the OTBM composite is less sensitive to practice effects because cutoffs are not used when the OTBM is calculated.

The problems associated with interpreting change on the deficit-based composites are also seen when inspecting the cutoffs for reliable change presented in Table 6. The cutoffs for the OTBM composites are straightforward to interpret, but the cutoffs for the deficit-based composites are in many cases less meaningful. For example, on the paper-and-pencil #≤5th %tile composite in the community control group, for an individual participant to be among the 20% with greatest improvement (i.e., the 80th percentile), the change from the 2-week to 3-month assessments must be >1. However, a change >1 is also required for being among the 10% with greatest improvement (i.e., the 90th percentile). Similarly, looking at the other tail of the distribution, where individuals who decline in performance are found, both the 10th and the 20th percentile correspond to a change score of zero. A closer inspection of this composite in the community control group shows that, out of 63 participants, one had a change score of 2 (i.e., a decline, because a higher number indicates worse performance on the deficit-based composites), four had a change score of 1, 44 had a change score of zero, 11 had a change score of −1 (i.e., an improvement), and three had a change score of −2. Thus, the range of change scores on this composite is narrow (i.e., there are only five different scores) and many participants have the same score. Because many participants share the same change score, most change scores corresponds not to one, but a range of percentiles. For example, the 44 participants with a change score of zero dominate the distribution of change scores (i.e., their ranks are from rank 6 to rank 49) and a change score of zero on this composite means that the participant is somewhere between the 9 and 77th percentile, indicating that this composite may lack sufficient sensitivity to detect change among most individuals.

Previous research on multivariate base rates has shown that among healthy individuals, the likelihood of obtaining at least one low score increases with the number of tests included in the test battery (26–33, 39). This is also the finding in the present study, in that the base rates of low scores were higher when all tests were considered, compared to the separated composites for the paper-and-pencil and CANTAB batteries. Thus, evaluating change on deficit-based composites may be more suitable on large test batteries, where more variability in scores is likely. Further, on the deficit-based composites, there is a general trend of higher test-retest correlation with higher cutoffs: the #<50th %tile composite had a higher test-retest correlation than the #≤16th %tile composite, which had a higher test-retest correlation than the # ≤ 5th %tile composite. Taken together, these findings indicate that on deficit-based composites, there might be a tradeoff between acceptable test-retest reliability, the number of tests in the neuropsychological battery, and the cutoff chosen to define a low score (i.e., scores at or below the 5 or 16th percentile).

This study is part of a research program with the aim of identifying a cognitive composite score suitable for TBI research and clinical trials. So far, the composites have been evaluated on healthy participants and patients with MTBI on the CENTER-TBI neuropsychological test battery, the Automated Neuropsychological Assessment Metrics (Version 4) Traumatic Brain Injury Military (ANAM4 TBI-MIL), and on the Delis-Kaplan Executive Function System (D-KEFS) (7–9). Because MTBI-related cognitive deficits often are subtle (44), diminish rapidly over time, and they might only be present in a small subgroup, identifying a sensitive composite is important, but challenging, in this patient population. The present sample consisting of patients with MTBI that are in the milder end of MTBI (i.e., the vast majority were non-hospitalized), had few, if any, cognitive deficits at their first assessment (9), and this constitutes a limitation of the present study as we cannot conclude with certainty which of the composites are most sensitive to change after TBI. Future studies should evaluate the composites in samples where cognitive deficits are likely, such as in the acute phase after MTBI, or in patients with moderate-to-severe TBI. In such samples, it is possible that the deficit-based composites would have better capacity to detect change. Further, the zero-inflation of the composite score distribution lead to non-normality, which limited the statistical methods available to calculate reliable change. Several methods for calculating reliable change exist (46), but because of the non-normal data, only the natural distribution of change scores was used in the present study. For example, when calculating the reliable change index (RCI), the standard deviations from the two assessments are used to calculate the standard error of measurement (47), and in zero-inflated distribution, the standard deviation is a poor and biased measure of dispersion.

Improvements were largest on the OTBM composite and this composite had the highest test-retest reliability. Although these findings appear to favor the use of OTBM in research and clinical trials that analyze change trajectories, more research is necessary to replicate these findings in different test batteries, in more severely injured samples, who have varying degrees of cognitive impairment, and assess the association between these composites and functional outcomes in people with moderate or severe TBIs. For clinical trials that compare two or more groups at a single time point (e.g., post-treatment), the OTBM may be less advantageous. Lastly, these candidate composite scores reflect a developing body of research on the best methods to summarize cognitive test data for use in research and clinical trials, but they are not exhaustive and other composites might ultimately prove to be preferred.

## Data Availability Statement

The datasets generated for this study are available on request to the corresponding author.

## Ethics Statement

The studies involving human participants were reviewed and approved by the regional committee for research ethics (REK 2013/754). The patients/participants provided their written informed consent to participate in this study.

## Author Contributions

JS, JK, NS, and GI designed the study. JS performed the statistical analyses. TS secured funding for the larger parent study and directed that study. JS, RK, and TS collected the data. JS and GI drafted the manuscript. All authors critically reviewed the manuscript. All authors read and approved the last version of this manuscript.

## Conflict of Interest

GI served as a scientific advisor for BioDirection, Inc., Sway Operations, LLC, and Highmark, Inc. He had a clinical and consulting practice in forensic neuropsychology, including expert testimony, involving individuals who have sustained mild TBIs. He had received research funding from several test publishing companies, including ImPACT Applications, Inc., CNS Vital Signs, and Psychological Assessment Resources (PAR, Inc.). He received royalties from one neuropsychological test (WCST-64; PAR, Inc.). He had received research funding as a principal investigator from the National Football League, and salary support as a collaborator from the Harvard Integrated Program to Protect and Improve the Health of National Football League Players Association Members. He acknowledged unrestricted philanthropic support from ImPACT Applications, Inc., the Heinz Family Foundation, and the Mooney-Reed Charitable Foundation. The remaining authors declare that the research was conducted in the absence of any commercial or financial relationships that could be construed as a potential conflict of interest.
